# Histone deacetylation regulates de novo shoot regeneration

**DOI:** 10.1093/pnasnexus/pgad002

**Published:** 2023-01-06

**Authors:** Haruka Temman, Takuya Sakamoto, Minoru Ueda, Kaoru Sugimoto, Masako Migihashi, Kazunari Yamamoto, Yayoi Tsujimoto-Inui, Hikaru Sato, Mio K Shibuta, Norikazu Nishino, Tomoe Nakamura, Hiroaki Shimada, Yukimi Y Taniguchi, Seiji Takeda, Mitsuhiro Aida, Takamasa Suzuki, Motoaki Seki, Sachihiro Matsunaga

**Affiliations:** Department of Applied Biological Science, Faculty of Science and Technology, Tokyo University of Science, 2641 Yamazaki, Noda, Chiba 278-8510, Japan; Department of Applied Biological Science, Faculty of Science and Technology, Tokyo University of Science, 2641 Yamazaki, Noda, Chiba 278-8510, Japan; Plant Genomic Network Research Team, RIKEN Center for Sustainable Resource Science, 1-7-22 Suehiro, Tsurumi, Yokohama, Kanagawa 230-0045, Japan; Plant Epigenome Regulation Laboratory, RIKEN Cluster for Pioneering Research, 2-1 Hirosawa, Wako, Saitama 351-0198, Japan; Department of Applied Biological Science, Faculty of Science and Technology, Tokyo University of Science, 2641 Yamazaki, Noda, Chiba 278-8510, Japan; Department of Integrated Biosciences, Graduate School of Frontier Sciences, The University of Tokyo, 5-1-5 Kashiwanoha, Kashiwa, Chiba 277-8562, Japan; Department of Integrated Biosciences, Graduate School of Frontier Sciences, The University of Tokyo, 5-1-5 Kashiwanoha, Kashiwa, Chiba 277-8562, Japan; Department of Integrated Biosciences, Graduate School of Frontier Sciences, The University of Tokyo, 5-1-5 Kashiwanoha, Kashiwa, Chiba 277-8562, Japan; Department of Integrated Biosciences, Graduate School of Frontier Sciences, The University of Tokyo, 5-1-5 Kashiwanoha, Kashiwa, Chiba 277-8562, Japan; Academic Assembly (Faculty of Science), Yamagata University, Kojirakawa, Yamagata 990-8560, Japan; Graduate School of Life Science and Systems Engineering, Kyushu Institute of Technology, 2-4 Hibikino, Wakamatsu-ku, Kitakyushu-shi, Fukuoka 808-0196, Japan; Plant Genomic Network Research Team, RIKEN Center for Sustainable Resource Science, 1-7-22 Suehiro, Tsurumi, Yokohama, Kanagawa 230-0045, Japan; Department of Biological Science and Technology, Tokyo University of Science, 6-3-1 Niijuku, Katsushika-ku, Tokyo 125-8585, Japan; Department of Biological Science and Technology, Tokyo University of Science, 6-3-1 Niijuku, Katsushika-ku, Tokyo 125-8585, Japan; School of Science and Technology, Kwansei Gakuin University, 2-1 Gakuen, Sanda, Hyogo 669–1337, Japan; Graduate School of Life and Environmental Sciences, Kyoto Prefectural University, Shimogamo Hangi-cho, Sakyo-ku, Kyoto 60-8522, Japan; Biotechnology Research Department, Kyoto Prefectural Agriculture Forestry and Fisheries Technology Centre, 74 Kitaina Yazuma Oji, Seika, Kyoto 619-0244, Japan; International Research Organization for Advanced Science and Technology, Kumamoto University, 2-39-1 Kurokami, Chuo-ku, Kumamoto 860-8555, Japan; International Research Center for Agricultural and Environmental Biology, Kumamoto University, 2-39-1 Kurokami, Chuo-ku, Kumamoto 860-855, Japan; College of Bioscience and Biotechnology, Chubu University, 1200 Matsumoto-cho, Kasugai, Aichi 487-8501, Japan; Plant Genomic Network Research Team, RIKEN Center for Sustainable Resource Science, 1-7-22 Suehiro, Tsurumi, Yokohama, Kanagawa 230-0045, Japan; Plant Epigenome Regulation Laboratory, RIKEN Cluster for Pioneering Research, 2-1 Hirosawa, Wako, Saitama 351-0198, Japan; Department of Integrated Biosciences, Graduate School of Frontier Sciences, The University of Tokyo, 5-1-5 Kashiwanoha, Kashiwa, Chiba 277-8562, Japan

**Keywords:** shoot regeneration, histone deacetylation, epigenetics, transdifferentiation

## Abstract

During de novo plant organ regeneration, auxin induction mediates the formation of a pluripotent cell mass called callus, which regenerates shoots upon cytokinin induction. However, molecular mechanisms underlying transdifferentiation remain unknown. Here, we showed that the loss of *HDA19*, a histone deacetylase (HDAC) family gene, suppresses shoot regeneration. Treatment with an HDAC inhibitor revealed that the activity of this gene is essential for shoot regeneration. Further, we identified target genes whose expression was regulated through HDA19-mediated histone deacetylation during shoot induction and found that *ENHANCER OF SHOOT REGENERATION 1* and *CUP-SHAPED COTYLEDON 2* play important roles in shoot apical meristem formation. Histones at the loci of these genes were hyperacetylated and markedly upregulated in *hda19*. Transient *ESR1* or *CUC2* overexpression impaired shoot regeneration, as observed in *hda19*. Therefore, HDA19 mediates direct histone deacetylation of *CUC2* and *ESR1* loci to prevent their overexpression at the early stages of shoot regeneration.

Significance Statement We found that the de novo shoot regeneration from a pluripotent cell mass called callus is regulated by a histone deacetylase family gene, *HDA19*. The two key genes responsible for shoot apical meristem formation are directly deacetylated by HDA19 at the early stages of shoot regeneration.

## Introduction

Plants possess high pluripotent competency (1, 2), enabling de novo shoot organogenesis from explants using in vitro tissue culture techniques, which have been extensively used in various studies including those on transgenic breeding and propagation of economically important traits. The knowledge of molecular mechanisms underlying de novo organogenesis would not only enhance our understanding of plant differentiation but also help improve tissue culture techniques for agricultural and horticultural applications. During de novo shoot regeneration, first, a mass of undifferentiated pluripotent cells called callus is formed from an explant following incubation on an auxin-rich callus-inducing medium (CIM) (3, 4). Second, shoots are regenerated from the callus following incubation on a cytokinin-rich shoot-inducing medium (SIM) (3). During callus formation, plants are reprogrammed to acquire pluripotent competency in the middle layer of callus, which is required for shoot regeneration (3, 5, 6). Following incubation on SIM, shoot apical meristems (SAMs) are formed from the callus (7, 8).

The molecular pathway of de novo shoot regeneration has been gradually revealed (1, 9, 10). During shoot induction, the transcription factors PLETHORA (PLT) 3, PLT5, and PLT7 upregulate *CUP-SHAPED COTYLEDON* (*CUC*) *2* and the transcription factor ENHANCER OF SHOOT REGENERATION 1/DORNRÖSCHEN (ESR1/DRN) upregulates *CUC1* (11, 12). At the early stages of shoot induction, spatial expression of *CUC1* and *CUC2* (13, 14) determines the location of SAM on the callus (13, 14). At subsequent stages, WUSCHEL (WUS) functions as a core transcription factor regulating the development of SAMs from shoot progenitors on the callus (14, 15). *WUS* expression is activated by cytokinin signaling transcription factors, including type-B ARABIDOPSIS RESPONSE REGULATOR 1 (ARR1), ARR2, ARR10, and ARR12 (16, 17).

Moreover, the expression of genes involved in shoot regeneration is epigenetically regulated through chromatin modification and transcriptional activity (18). For instance, at the early stages of shoot regeneration, *WUS* expression is repressed through DNA methylation via DNA methyltransferases (19–21). Furthermore, during shoot regeneration, histone modifiers, such as KRYPTONITE, JUMONJI14, and HISTONE ACETYLTRANSFERASE OF CBP FAMILY 1, and histone methyltransferases of the polycomb repressive complex 2 have been implicated in dynamic *WUS* expression through the regulation of histone methylation and acetylation status at the *WUS* locus (16, 19). The ARABIDOPSIS TRITHORAX-RELATED 2–ARR1 complex activates the type-A ARR genes *ARR5* and *ARR7* through H3K36me3 methylation, thereby repressing cytokinin signaling and inhibiting *WUS* expression at an inappropriate timing (22). Additionally, some histone modifiers play crucial roles in the acquisition of shoot regenerative competency during callus formation. Upon incubation on CIM, GENERAL CONTROL NONREPRESSED PROTEIN 5—a histone acetyltransferase (HAT) of the GNAT/MYST superfamily 1—activates the expression of *WUSCHEL RELATED HOMEOBOX 5* (*WOX5*), *WOX14, SCARECROW, PLT1*, and *PLT2*, which establish callus pluripotency (23). Further, LYSINE-SPECIFIC DEMETHYLASE 1-LIKE3-mediated H3K4me2 demethylation of shoot regeneration-related genes, such as *CBL-INTERACTING PROTEIN KINASE 23, NADH DEPENDENT GLUTAMATE SYNTHASE 1*, and *UBIQUITIN-PROTEIN LIGASE 4*, during callus formation is required for their activation in response to shoot induction (24). Thus, epigenetic regulation plays a pivotal role throughout shoot regeneration, although the precise mechanism remains to be fully elucidated.

Here, we focus on a counterpart of HAT, histone deacetylase (HDAC), which represses gene expression by catalyzing histone H3 and H4 deacetylation (25, 26). Among HDACs in *Arabidopsis thaliana*, we showed that the function of HISTONE DEACETYLASE 19 (HDA19) is essential for shoot regeneration upon incubation on SIM. Further, we found that HDA19 directly deacetylates histones at *CUC2* and *ESR1* loci, preventing their overexpression upon shoot induction. In addition, transgenic plants transiently overexpressing *CUC2* or *ESR1* during incubation on SIM phenocopied *hda19* in terms of shoot regeneration efficiency. Based on these results, we propose that although *CUC2* and *ESR1* induction is essential for SAM formation at the early stages of shoot regeneration, their expression level must be stringently regulated, and HDA19-mediated histone deacetylation plays a crucial role in this process.

## Results

### HDA19 is required for de novo shoot regeneration from callus

**Fig. 1.**
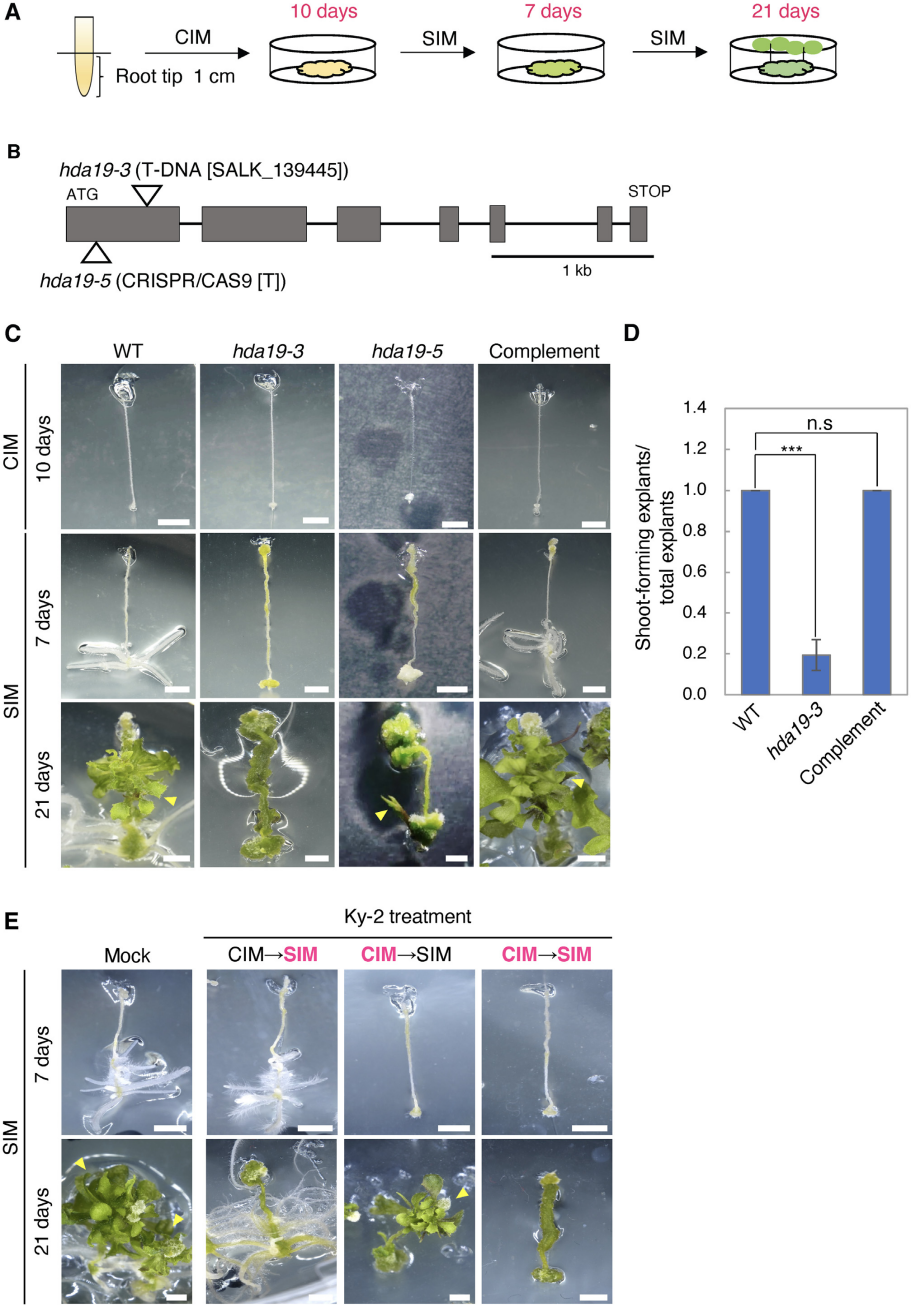
Shoot regeneration phenotypes in *hda19* and WT under suppressed HDAC activity. (A) Schematic of de novo shoot regeneration. Root tip (0 to 1 cm root explants) were excited from seedlings at 7 days after germination and incubated on CIM for 10 days, followed by SIM for 21 days. Phenotypes were observed at 10 days on CIM, 7 days on SIM, and 21 days on SIM. (B) Gene structure of *HDA19* and the sites of T-DNA insertion and T-residue insertion using CRISPR/CAS9. Boxes: exons; bars: introns. (C) Phenotypes of WT, *hda19-3, hda19-5*, and a complementation line (complement, *HDA19p-HDA19g-sGFP* in *hda19-3*) at 10 days on CIM, 7 days on SIM, and 21 days on SIM. Scale bars = 2 mm. Arrow heads: regenerated shoots. (D) Shoot regeneration rate (the ratio of shoot-forming explants to the total explants) in WT, *hda19-3*, and complement explants at 21 days on SIM. Results are presented as means ± SE of at least three independent experiments (***P < 0.001, Student’s *t*-test). (E) Phenotypes of WT explants at 7 and 21 days on SIM with 1 μM Ky-2 on CIM and/or SIM. Ky-2 treatment is indicated by magenta letters. Scale bars = 2 mm. Yellow arrowheads indicate regenerated shoots.

To investigate the roles of HDACs in de novo shoot regeneration, we performed shoot regeneration assays using root explants excised from regions at 0 to 1 cm from the root tip (0 to 1 cm root explants) of wildtype (WT) and *hdac* mutant plants (Fig. S1). We examined *hdac* mutants, including *hda6, hda9, hda10, hda14, hda17, hda18, hda19*, and *histone deacetylase 2b* (*hd2b*), and found that shoot regeneration was suppressed only in *hda19*, indicating that HDA19 is specifically involved in shoot regeneration from calli (Fig. S1). On day 10 of CIM incubation, there were no differences in callus formation between WT and *hda19* (*hda19-3*: T-DNA insertion allele; *hda19-5*: insertion allele using CRISPR/Cas9) (Fig. 1B and C). In contrast, on day 21 of SIM incubation, the shoot regeneration efficiency of root explants from WT and *hda19* calli was 100% and 20%, respectively (Fig. 1C and D). Similarly, shoot regeneration from different tissues, such as the hypocotyl, first leaf, and petal of *hda19* explants was suppressed (Fig. S2). Moreover, in *hda19* calli from which shoot regeneration was confirmed, the number of regenerated shoots was extremely low (one or two) and their morphology was abnormally thin and long leaf-like (Fig. 1C). This reduction in the shoot regeneration rate of *hda19*-*3* (hereafter referred to as *hda19*) was recovered by *HDA19-GFP* expression under the control of its own promoter (*HDA19p*::*HDA19g-sGFP*) (Fig. 1C and D). Taken together, these results demonstrate that HDA19 is required for de novo shoot regeneration from calli.

Next, to verify that the observed phenotype of *hda19* explants was due to defective HDA19 activity, we examined whether treatment with Ky-2 (27, 28), an inhibitor of HDAC family RPD3-like class I [e.g., HDA6, HDA7, HDA9, and HDA19 (29)], produced a phenotype similar to *hda19*. WT root explants were incubated on CIM and SIM with or without Ky-2, and the shoot regeneration phenotype was evaluated. During both CIM and SIM incubation, Ky-2 treatment suppressed shoot regeneration (Fig. 1E). Furthermore, shoot regeneration was observed in the presence of Ky-2 only during CIM incubation, whereas it was suppressed in the presence of Ky-2 only during SIM incubation (Fig. 1E). These results suggest that the HDAC activity of HDA19 during SIM incubation plays a pivotal role in shoot regeneration.

**Fig. 2.**
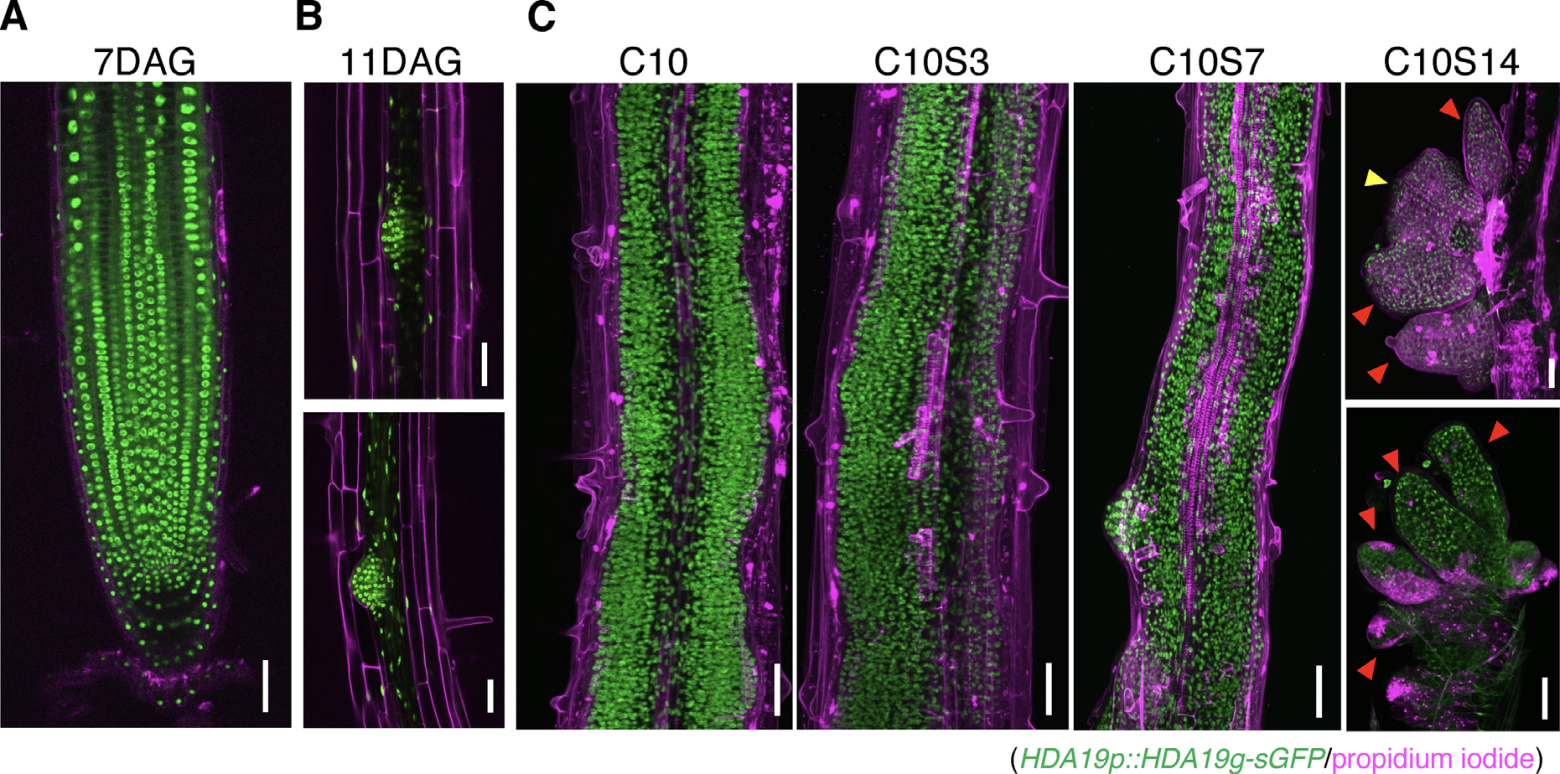
Expression pattern of HDA19 in roots and callus during shoot induction. (A to C) Expression pattern of the HDA19 translational reporter (*HDA19p::HDA19g-sGFP*, shown in green) in (A) root apical meristem at 7 days after germination (7 DAG), (B) LRP at 11 DAG, and (C) calli at 10 days on CIM (C10) and 3, 7, and 14 days on SIM (C10S3, C10S7, and C10S14, respectively). Cell outlines were visualized using propidium iodide (PI) staining (shown in magenta). Images in (A) and (B) represent a single optical section. Images in (C) represent *z*-projections. Scale bars = 45 μm. A yellow arrowhead indicates a developing SAM, and red arrowheads indicate leaf primordia.

Finally, we examined the expression pattern of HDA19 during shoot regeneration in root explants using a transgenic line expressing *HDA19p::HDA19g-sGFP* (*HDA19-GFP*). Imaging analysis showed that under normal growth conditions,*HDA19-GFP* was mainly localized in the nuclei and was expressed in all tissues at the root tip and at two distinct stages of lateral root primordia (LRP) (Fig. 2A and B). Just before shoot induction (10 days on CIM: C10), *HDA19-GFP* expression was observed in all callus tissues, except epidermal cells. This expression pattern of *HDA19-GFP* was also seen in calli at 3 and 7 days after shoot induction following the incubation on CIM for 10 days (C10S3 and C10S7). At C10S14, *HDA19-GFP* was expressed in and around the SAM; however, it was weakly expressed in callus (Fig. 2C).

### HDA19 affects gene expression by regulating histone acetylation status during shoot induction

**Fig. 3.**
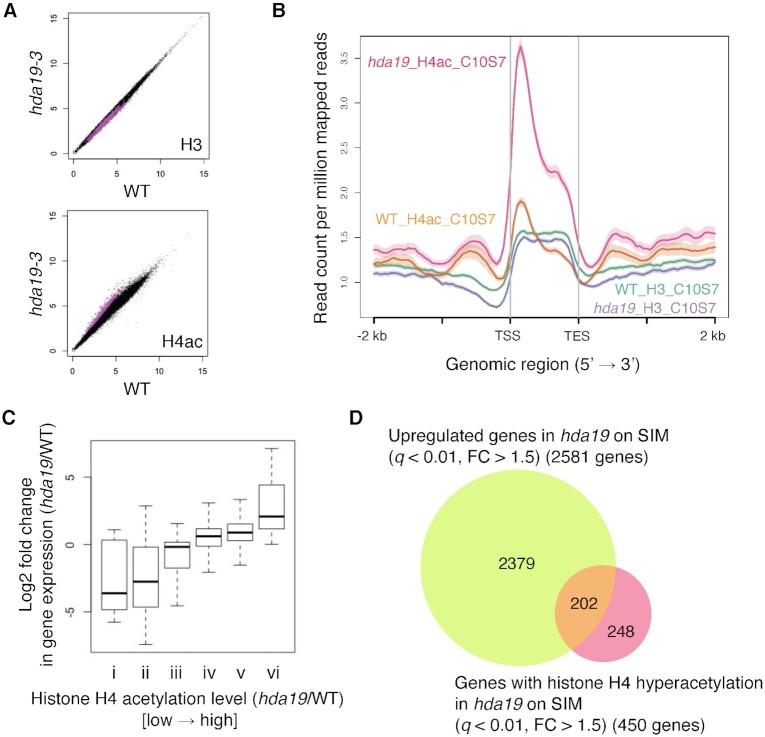
Histone acetylation and gene expression levels in *hda19* during shoot induction. (A) Histone H3 and acetylated histone H4 levels in *hda19* compared with WT at 7 days on SIM. Each black dot represents the square root of the count of mapped reads of all genes. Magenta dots indicate 450 genes with histone H4 hyperacetylation in *hda19* compared with WT (*q* < 0.01, FC > 1.5). (B) Positional profiles of histone H3 and acetylated histone H4 in WT and *hda19* on genic region (gene plus 2 kb sequence up- and downstream) of 450 genes with histone H4 hyperacetylation in *hda19* on SIM shown in (A). (C) Association between histone acetylation and gene expression level in *hda19*. Based on differences in histone H4 acetylation level between WT and *hda19* [log2(RPM _*hda19*/RPM_WT)] at 7 days on SIM shown in (A), genes were divided into six groups (*x*-axis). Gene expression fold-changes between WT and *hda19* [log2(RPM_*hda19*/RPM_WT)] at 7 days on SIM were calculated and plotted for group. (D) Venn diagram of genes with histone H4 hyperacetylation in *hda19* on SIM shown in (A) and upregulated genes on SIM in *hda19* compared with WT (*q* < 0.01, FC > 1.5).

To clarify the role of HDA19-mediated histone deacetylation during shoot regeneration, we performed integrated analysis with RNA-seq and chromatin immunoprecipitation, followed by sequencing (ChIP-seq), on callus incubated on SIM for 7 days after 10 days of incubation on CIM (Data S1 and S2). HDA19 affects the acetylation status of histones H4 and H3 (29, 30). ChIP-seq of acetylated histone H4 (H4ac) showed that the histone acetylation level of 450 genes was significantly elevated (*q* < 0.01, FC > 1.5) in *hda19* compared with that in WT (Fig. 3A, Data S2). Positional profiles of H4ac in the genic region (gene body plus 2 kb sequences up- and downstream) of 450 hyperacetylated genes revealed higher H4ac levels throughout the gene-coding region in *hda19* than in WT (Fig. 3B). Meanwhile, histone H3 accumulation and distribution profiles in the genic region of hyperacetylated genes were comparable between WT and *hda19* (Fig. 3A and B). We next analyzed whether the histone acetylation and gene expression levels were positively correlated in *hda19*. We classified genes into six groups (i to vi, low to high) based on the histone acetylation level in *hda19* relative to that in WT and compared their expression between WT and *hda19* (Fig. 3C). Histone acetylation level was correlated with gene expression level in *hda19* (Fig. 3C). We identified 202 candidate target genes of HDA19, whose expression (*q* < 0.01, FC > 1.5) and histone acetylation levels were increased in *hda19* compared with those in WT (Fig. 3D, Data S1).

### HDA19 regulates shoot regeneration through *ESR1* and *CUC2* repression

**Fig. 4.**
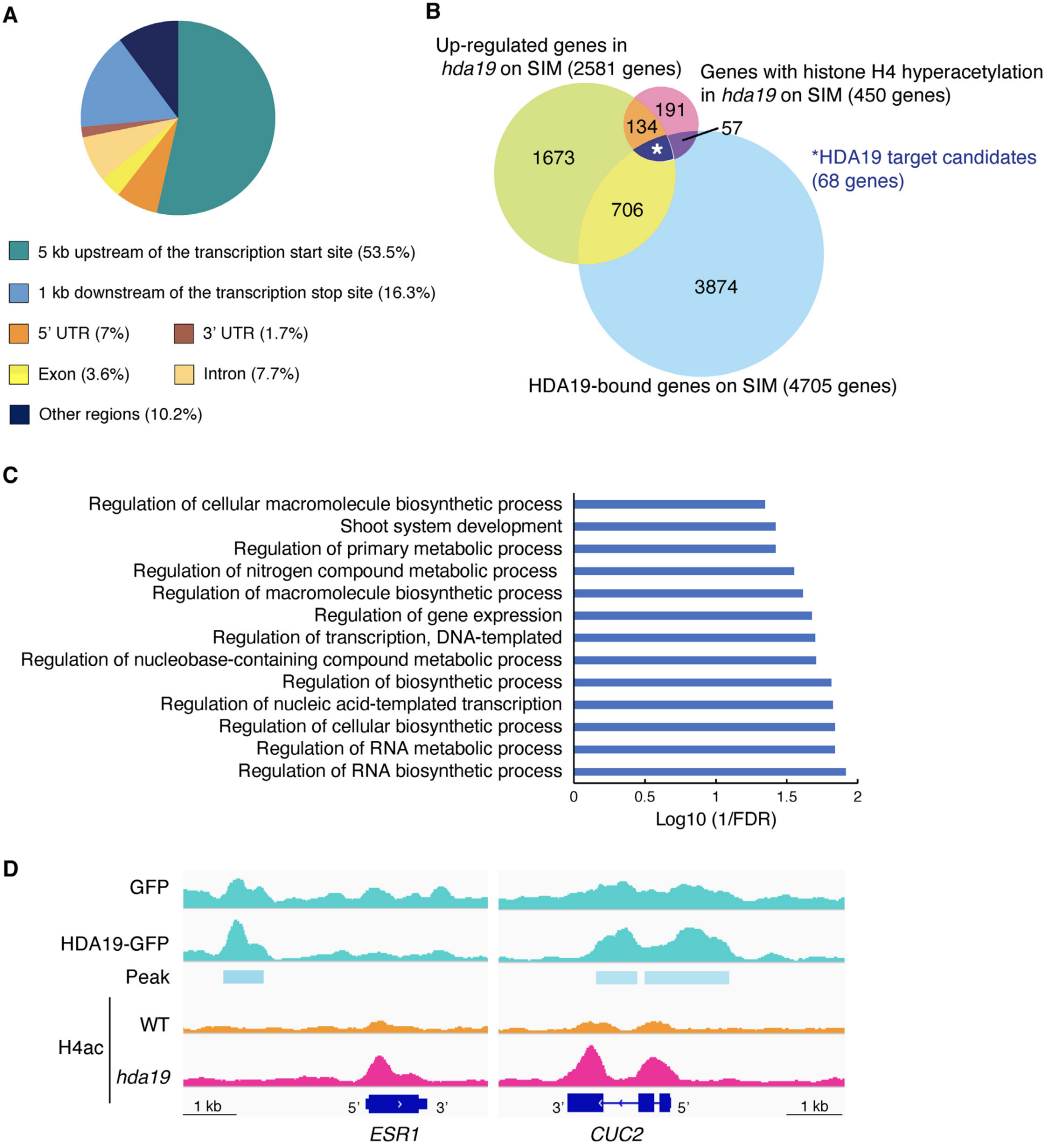
Identification of HDA19 target genes involved in shoot regeneration. (A) Pie chart showing the percentage of gene regions to which HDA19 could bind. HDA19 binding sites were determined using MACS2 peak calling (*q* < 0.001). A total of 8,823 peaks were analyzed. (B) Venn diagram of genes with histone H4 hyperacetylation in *hda19* on SIM (*q* < 0.01, FC > 1.5), upregulated in *hda19* on SIM (*q* < 0.01, FC > 1.5), bound to HDA19 on SIM [*q* < 0.001, −log10(*P-*value) > 20]. Genes that met all conditions were considered HDA19 target candidates. (C) Gene Ontology analysis of HDA19 target candidates (FDR < 0.05). (D) Distribution of acetylated histone H4 and HDA19 binding sites around the *ESR1* and *CUC2* loci at 7 days on SIM. Blue boxes indicate exons and blue lines indicate introns.

Next, we investigated the binding of HDA19 at 7 days on SIM using ChIP-seq in an *hda19* line harboring *HDA19-GFP* and an anti-GFP antibody. We identified 8,823 HDA19-binding sites in the genome and found that HDA19 was bound to the upstream regions of majority of the genes (Fig. 4A, Data S4). From these, we identified 68 genes whose expression and histone acetylation levels were increased in *hda19* compared with those in WT and to which HDA19 bound (Fig. 4B, Data S1). These were considered candidate genes that may be directly regulated by HDA19 and implicated in conferring the shoot regeneration phenotype of *hda19*. In Gene Ontology analysis of the candidates, genes involved in shoot system development were significantly enriched (FDR < 0.05) (Fig. 4C).

Of the 68 genes, we focused on two transcription factors responsible for shoot formation: *ESR1* and *CUC2* (31, 32). HDA19-binding sites were detected in the upstream regions of *CUC2* and *ESR1* and the gene-coding region of *CUC2* (Fig. 4D). Therefore, HDA19 may directly reduce the histone acetylation level in both gene-coding regions by binding at the periphery of the genic regions of *CUC2* and *ESR1*.

**Fig. 5.**
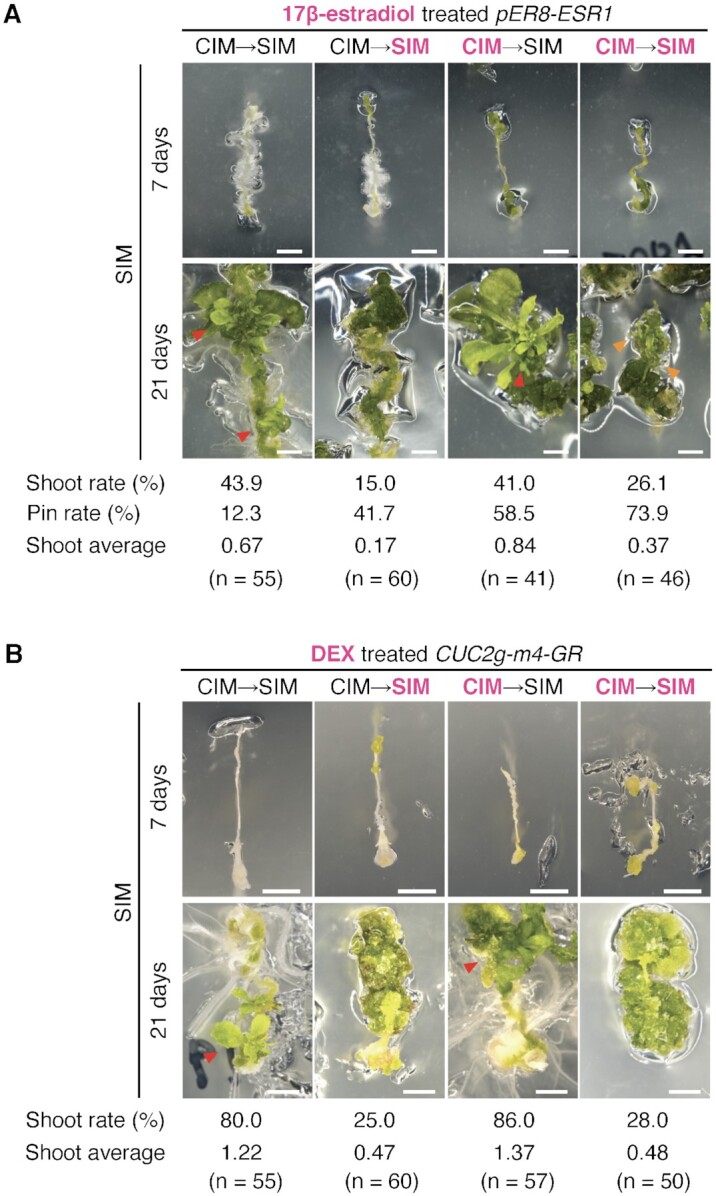
Effects of *ESR1* and *CUC2* overexpression on the shoot regeneration phenotype. (A) Shoot regeneration phenotypes of *ESR1* conditional overexpression line *pER8-ESR1* explants at 7 and 21 days on SIM with 5 μM 17β-estradiol on CIM and/or SIM. 17β-Estradiol treatment is indicated by magenta letters. (B) Shoot regeneration phenotypes of *CUC2* conditional overexpression line *CUC2g-m4-GR* explants at 7 and 21 days on SIM with 1 μM DEX on CIM and/or SIM. DEX treatment is indicated by magenta letters. Shoot rate is the ratio of normal shoot-forming explants to the total explants. Pin rate is the ratio of shoot-forming explants with a pin-like leaf to the total shoot-forming explants. Shoot average is the number of shoots per explant tested. Scale bars = 2 mm. Red and orange arrowheads indicate regenerated shoots and a pin-like leaf, respectively.

Next, to evaluate whether the *hda19* phenotype during shoot regeneration could be attributed to the increased expression of *ESR1* and *CUC2*, we conditionally overexpressed *ESR1* or *CUC2* and observed the resulting phenotypes (Fig. 5). *ESR1* was transiently induced using *pER8-ESR1*, which induced *ESR1* through 17β-estradiol application (31). *CUC2* was transiently induced using *CUC2g-m4-GR*, which induced *CUC2* through dexamethasone (DEX) application. *CUC2g-m4-GR* carries a mutation in the miR164 target sequence, which prevents *CUC2* mRNA degradation to induce its overexpression (33). Following the incubation of *pER8-ESR1* or *CUC2g-m4-GR* root explants on CIM for 10 days, shoot regeneration was analyzed at 7 and 21 days on SIM with/without the inducer. Expectedly, *ESR1* or *CUC2* overexpression through the application of the respective inducers on both CIM and SIM or SIM alone resulted in defects in shoot regeneration, similar to that in *hda19* (Fig. 5A and B). Meanwhile, *ESR1* or *CUC2* overexpression in CIM alone did not affect shoot regeneration. Furthermore, we noted a pin-like leaf morphology of regenerated shoots in all conditions under *ESR1* overexpression (Fig. 5A).

Therefore, HDA19 may be involved in shoot regeneration during SIM incubation by directly regulating the histone acetylation levels and, ultimately, the expression levels of *ESR1* and *CUC2*. The comparable expression levels of *ESR1* and *CUC2* in a complement line of *hda19* and in WT at C10S7 support this hypothesis (Fig. S3).

### Lack of localized expression of *ESR1* and *CUC2* may inhibit shoot regeneration in *hda19*

**Fig. 6.**
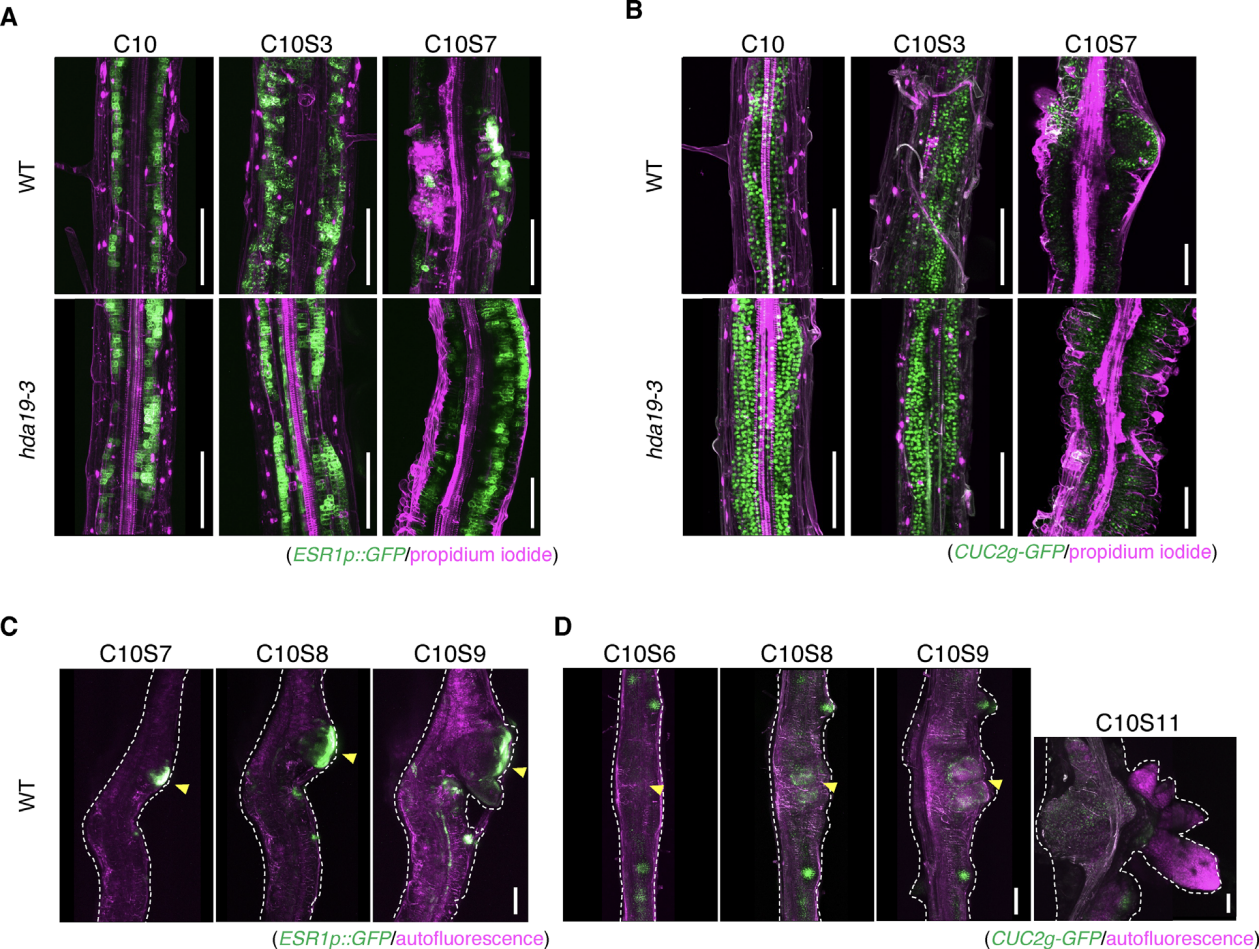
Dynamics of *ESR1* and *CUC2* expression during shoot induction in *hda19*. Expression patterns of (A) *ESR1p::GFP* and (B) *CUC2g-GFP* during shoot induction in WT and *hda19*. Time-lapse images of *ESR1p::GFP* (C) and *CUC2g-GFP* (D) expression. The same explants were observed at each time point. GFP fluorescence is shown in green, and the outline of PI-stained cells or autofluorescence is shown in magenta. The images represent *z*-projections. Scale bar = 100 µm. Arrowheads indicate developing SAMs.

To investigate why *ESR1* or *CUC2* overexpression inhibited shoot regeneration, we introduced the respective reporters *ESR1p::GFP* and *CUC2g-GFP* into *hda19* through crossing and observed fluorescent signals from callus formation to shoot regeneration. Both transgenes were considered to be regulated in the same manner as endogenous genes because *ESR1p::GFP* and *CUC2g-GFP* have upstream regions of approximately 4.8 and 3.1 kb, respectively, which overlap with the upstream HDA19-binding regions of approximately 2.8 and 1 kb, respectively (Data S3). In WT, *ESR1p::GFP* fluorescence signals were detected throughout the root explant at the time of callus formation (C10) and early stages of shoot regeneration (C10S3) (Fig. 6A). Subsequently, *ESR1p::GFP* fluorescence signals were concentrated at several locations of WT root explants at 7 days of incubation on SIM (Fig. 6A). Meanwhile, in *hda19, ESR1p::GFP* fluorescence signals were observed throughout the root explants, similar to that in WT, after 3 days of incubation on SIM, although the signals were stronger and expressed in more cells of root explants in the mutant (Fig. 6A). Even on day 7 of incubation on SIM, *ESR1p::GFP* fluorescence signals were observed throughout *hda19* root explants, although no localized expression of *ESR1p::GFP* fluorescence was observed in calli (Fig. 6A).

Next, in WT, the fluorescence signals of *CUC2g-GFP* were observed throughout the root explants after 3 days of incubation on SIM and accumulation of fluorescence was noted after 7 days of incubation on SIM (Fig. 6B). In *hda19*, the fluorescence signals of *CUC2g-GFP* were detected throughout the root explants, similar to that in WT after 3 days of incubation on SIM, although accumulation of fluorescence was not observed after 7 days of incubation on SIM (Fig. 6B). To examine the link between the localized expression of *ESR1* and *CUC2* and regeneration of SAM, we performed time-lapse observations of *ESR1p::GFP* and *CUC2g-GFP* fluorescence in WT (Fig. 6C and D). SAM was regenerated from the regions of fluorescence accumulation in root explants (Fig. 6C and D). Therefore, the inhibition of shoot regeneration in *hda19* may be caused by the lack of localized expression of *ESR1* and *CUC2* during SAM regeneration. This hypothesis is further supported by the data using Ky-2. The inhibition of HDAC activity only during incubation on SIM increased the expression of *ESR1* and *CUC2* transcripts (Fig. S3) and was responsible for the lack of specific localization of *ESR1p::GFP* and *CUC2g-GFP* fluorescence at C10S7 as observed in *hda19* (Fig. S4).

## Discussion

In the present study, we showed that supplementing SIM with an HDAC inhibitor during incubation remarkably repressed shoot regeneration (Fig. 1E). In addition, at the early stage of shoot induction, *hda19* exhibited an increased level of histone acetylation in 202 genes, which also showed elevated expression (Fig. 3D). These results strongly suggest that the HDAC activity of HDA19 during incubation on SIM is essential for the repression of specific genes to establish proper shoot regeneration. However, the regulation of histone acetylation status by HDA19 does not appear to be involved in the acquisition of regenerative competency during callus development.

Similar to many HDACs (30, 34–40), HDA19 binds to the upstream regions of genes and mediates histone deacetylation primarily near the transcription start site (Fig. 4A). This property of HDA19 is consistent with previous reports that HDA19 regulates differentiation and stress responses by binding to the upstream and coding regions of genes (30, 35, 38, 41–43). In the case of shoot induction, the expression level of *WUS*, a core factor regulating SAM development in the later stages of shoot induction (14, 15), was equivalent in WT and *hda19* at C10S7 (Fig. S3), suggesting that HDA19 regulates genes that function before the stage of *WUS* activity. Intriguingly, we found that two essential genes for the early stage of shoot regeneration, namely *ESR1* and *CUC2* (31, 44, 45), were included in the 68 genes whose histone acetylation and expression were repressed via HDA19 binding (Fig. 4B and D). The expression of both these genes is elevated during the early stages of shoot regeneration, followed by decline (13, 31). In addition, optimal level of *ESR1* expression during shoot induction is essential; in explants constitutively overexpressing *ESR1*, shoot regeneration is inhibited and abnormal leaf morphology is observed (31). Accordingly, the inability to suppress the expression of these genes to optimal levels in the appropriate cells at the appropriate time of shoot regeneration is a possible cause of the *hda19* phenotype. In fact, constitutive *ESR1* or *CUC2* overexpression, at least during incubation on SIM, inhibited shoot regeneration (Fig. 5). Since the expression of 46 of the candidate targets of HDA19 was upregulated upon shoot induction, even in the WT (Fig. S5, Data S1: C10 vs. C10S7), HDA19 may be also responsible for regulating the expression of these genes to the correct level in response to shoot induction.

*CUC2* was expressed throughout the callus during its development, and upon transfer to SIM, its expression was progressively restricted to the zone of SAM emergence (14). Consistent with observations reported by Matsuo et al. (46), our image analysis results confirmed that the dynamics of *ESR1* expression in WT calli was similar to those of *CUC2* during shoot regeneration. However, such restricted expression patterns of both *CUC2* and *ESR1* were not detected in *hda19* (Fig. 6). Therefore, HDA19 likely represses *ESR1* and *CUC2* expression in cells other than the SAM-generating cells, thus contributing to their localized expression in callus upon shoot induction. Moreover, ESR1 upregulates the paralog of *CUC2, CUC1*, whose expression is also restricted to SAM-generating cells upon shoot induction (13, 14, 46). We found that *CUC1* expression was significantly enhanced in *hda19* incubated on SIM (Data S1), implying that HDA19 is indirectly implicated in regulating *CUC1* dynamics in calli. Since HDA19 was expressed throughout the callus, including SAM cells, even after transfer to SIM, we speculate that there are functional differences in HDA19 between SAM-generating and other cells, which may be shaped by other factors, such as HDA19 interacting proteins. Indeed, HDA19 interacts with an array of partners during various developmental processes and stress responses (30, 35, 38, 41–43).

Furthermore, the 68 HDA19 target genes included other regulators that may be involved in various shoot regenerative processes, such as cell proliferation, auxin biosynthesis, photosynthesis, and pluripotency-related gene regulation (Fig. S5, Data S1). One of them is the transcription factor *WOX11*, which is known to activate *LATERAL ORGAN BOUNDARIES DOMAIN16*(*LBD16*) and *WOX5* and is involved in the acquisition of pluripotency in callus cells (6, 23, 47–49). In WT, the expression of *WOX11* in calli was repressed upon transfer to SIM (23, 49) (Data S1). Therefore, the direct repression of *WOX11* expression by HDA19 may contribute to the differentiation of pluripotent callus cells into SAM-generating cells. However, expression levels of *WOX5* in *hda19* were less than that in WT in response to shoot induction (Data S1). In addition, the expression pattern of *WOX5p::GFPer* during shoot induction was similar between WT and *hda19* (Fig. S6). These observations suggest that defects in the repression of other unknown *WOX11* downstream may be a possible cause of the *hda19* phenotype.

In conclusion, we showed that conditional fine-tuning of the expression of multiple key developmental genes through *HDA19*-mediated epigenetic regulation is important for determining the cell fate and loci of SAM formation during early shoot regeneration. Shoot progenitor formation in callus occurs sparsely, from which the developmental program proceeds sequentially to regenerate the shoot. Therefore, clarifying the spatial and temporal control mechanisms by determining which cells use which differentiation program at what time in callus tissue will lead to the development of methods to improve regeneration efficiency. The discovery and analysis of interacting factors will help us understand the mechanisms by which HDA19 regulates the spatiotemporal patterns and expression levels of genes important for shoot regeneration.

## Materials and methods

### Plant materials and growth conditions

The following mutant alleles were used in the present experiment: *hda19-3* (SALK_139445), *hda19-5* (a genome-edited allele in Col-0) (29), *hda6* (*axe1-4*), *hda18* (SAIL_1289_F05), *hda9* (SALK_007123), *hda10* (SM_3_28667), *hda14* (SALK_144995), *hda17* (SALK_090088), and *hd2b-2* (a genome-edited allele in Col-0); all mutants are on a Col-0 background. Col-0 plants harboring *pER8-ESR1* (31) were used for conditional *ESR1* overexpression. Col-0 plants harboring *ESR1p::GFP* (50) and *WOX5p::GFPer* (51) were used for imaging analysis. Col-0 plants harboring *35Sp::GFP* (24) were used for ChIP-seq. The generation of marker lines, a *CUC2-*overexpressing line, and an *hd2b* mutant line is described below. Combinations of the *hda19-3* mutation and markers were generated by crossing. Plants homozygous for the mutation and markers were selected for genotyping and antibiotic treatment. Plants were grown in soil or MGRL medium (52) under a long-day (16 h light/8 h dark) photoperiod.

### Regeneration assays

Root explants (0 to 1 cm from the root tip) were excised from seedlings 7 days after germination and cultured on CIM containing Gamborg’s B-5 medium (Wako, Osaka, Japan) with 20 g L^−1^ glucose (Wako), 0.5 g L^−1^ MES (Wako), 1 × Gamborg’s vitamin solution (Sigma–Aldrich, St. Louis, MO, USA), 500 µg L^−1^ 2,4-D (Sigma–Aldrich), 50 µg L^−1^ kinetin (Sigma–Aldrich), and 0.8% gellan gum (Wako); the pH was adjusted to 5.7 using 1.0 M KOH. Continuous light was used for callus induction.

After culturing for 10 days on CIM, the explants were transferred to SIM containing Gamborg’s B-5 medium, 10 g L^−1^ sucrose, 0.5 g L^−1^ MES, 1 × Gamborg’s vitamin solution, 2 µg mL^−1^ trans-zeatin, 0.4 µg mL^−1^ indole-3-butyric acid, 1 µg mL^−1^ d-biotin, and 0.8% gellan gum; the pH was adjusted to 5.7 using 1.0 M KOH. Continuous light was used for shoot induction. For the chemical treatment, Ky-2, 17β-estradiol, or DEX were added to CIM or SIM.

After incubation on SIM for 21 days, the number of explants that regenerated shoots on all explants was evaluated. All phenotypic assays and microscopic observations were performed at least twice.

### Microscopic imaging

To observe GFP fluorescent marker lines, 0.1 mg mL^−1^ propidium iodide (PI) (Sigma–Aldrich) was applied to the samples prior to imaging for counterstaining the cell outlines.

Root explants at the late stages of callus induction and at all stages of shoot induction (10 days on CIM and 3 to 14 days on SIM) and the root tips and LRP of seedlings (7 and 11 days after germination, respectively) were observed using an Olympus FVMPE-RS multiphoton microscope with an XLPLN 25 × WMP2 (N.A. = 1.05, WD = 2.00 mm) water immersion objective lens (Olympus, Shinjuku, Tokyo, Japan). To detect GFP, PI, and autofluorescence signals, the laser setting was tuned to 920 nm (for GFP) with a fixed 1,040 nm wavelength (for PI and autofluorescence). All lights were reflected by an FV30-SDM-M mirror. Signals were collected using an FV30-FGR filter mounted in front of GaAsP-PMTs. The *z*-stacks were reconstructed into a projection view using ImageJ. More than 10 samples were imaged for each marker line to confirm that the observed patterns were representative of the respective markers.

### Generation of transgenic plants

The *hd2b-2* allele was generated by genome editing as described previously (53, 54). To express a single guide RNA (sgRNA) and the CRISPR/Cas9 protein, pZH_OsU3gYSA_FFCas9 and pUC_AtU6oligo vectors were used for targeted mutagenesis in HD2B. The primer pair (5′-ATTGGTGGTTTTGAGTGTGACTGT-3′/5′-AAACACAGTCACACTCAAAACCAC-3′) was used for sgRNA to target HD2B. HD2B mutagenesis in Col-0 plants resulted in the generation of the *hd2b-2* allele by inserting a thymine at 140 nt downstream from the HD2B translation initiation codon, inducing a nonsense mutation at 151 nt from the translational initiation codon.

To generate *HDA19p::HDA19g-sGFP*, a genomic DNA fragment (1.5 kb upstream of ATG to the stop codon) from *HDA19* was amplified using 5′-CACCGGTAAAGCTTAAGATGGAAGCATGTGC-3′ and 5′- CGGAGCAGGCGTTTCCTCCTAAAACA-3′ primers and cloned into pENTR/D-TOPO using the Gateway system (Invitrogen, Carlsbad, CA, USA). The *HDA19* genomic sequence was recombined upstream of *sGFP* in the pGWB504 (55) binary vector using the Gateway LR Clonase II Enzyme Mix (Invitrogen) to generate *HDA19p::HDA19g-sGFP*, which was used for plant transformation (*hda19-3* for reporter generation complementation experiments). The constructed plasmid was transformed into *Agrobacterium tumefaciens* (*Rhizobium radiobacter*) strain GV3101::pMP90 and then into *hda19-3* using the floral dip method (56). Homozygotes were selected using antibiotics and analyzed.

For *CUC2g-m4-GR*, the stop codon of *CUC2g-m4* (33) was replaced with the coding sequence of rat GR receptor domain (57) with the linker sequences AAGCTTATCGATACCGTCGACCTCGAC and TGACTCGAG at the 5′ and 3′ ends, respectively. The entire fragment was cloned into the binary vector pBIN50 (58). For *CUC2g-GFP*, a genomic fragment of *CUC2* spanning from the 3.1 kb promoter to the end of the coding sequence was amplified using 5′-GGGGACAACTTTGTATAGAAAAGTTGACTAGAGGAAGAGTTAAGAGATG-3′ and 5′-GGGGACTGCTTTTTTGTACAAACTTGCGTAGTTCCAAATACAGTCAAG-3′ primers and cloned into *pDONR P4-P1R* (Invitrogen) using the Gateway system. The *CUC2* terminator was amplified using 5′-GGGGACAGCTTTCTTGT ACAAAGTGGCATCACAAAAGAGGTGACTTATA-3′ and 5′-GGGGACAACTTTGTATAATAAAGTTGAAATCATCTAACCGAAGATTCG-3′ primers and cloned into *pDONR P2R-P3* (Invitrogen). The inserts in these two plasmids, together with the GFP coding sequence in pDONR207, were transferred to the pGWBmultisite vector (59) to generate *CUC2p::CUC2-GFP::CUC2ter* (*CUC2g-GFP*). The constructed plasmids were transformed into *Agrobacterium tumefaciens* strain GV3101::pMP90 and then into Col-0 using the floral dip method. Homozygotes were crossed with *hda19-3*.

### RNA-seq

Root explants derived from WT (Col-0) and *hda19-3* seedlings were collected on day 10 of CIM (C10) and day 7 of SIM (C10S7) incubation. Total RNA was isolated from the explants using the PureLink Plant RNA Reagent (Thermo Fisher Scientific, Waltham, MA, USA). The integrity of purified RNA was assessed using the 2100 Bioanalyzer (Agilent, Hachioji, Tokyo, Japan). The extracted RNA (1,000 ng) was used to construct a transcriptome library with TruSeq RNA Sample Preparation v.2 (Illumina, San Diego, CA, USA). Libraries were pooled and 36 to 86 bp single-read sequences were obtained using the NextSeq 500 sequencer (Illumina). Three independent biological replicates were analyzed for each genotype.

### RNA-seq data analysis

Quality-filtered reads were mapped onto the cDNA sequences of annotated genes and other transcripts of TAIR10 using Bowtie (60) with-all-best-strata settings (Data S4). Differentially expressed genes (DEGs) were identified using the edgeR package in R version 3.16.5 (61), treating biological triplicates as paired samples. Genes with adjusted *q* < 0.01 and fold change (FC) > 1.5 or < 0.66 in each comparison were identified as DEGs (Data S1). To calculate relative gene expression (*hda19*/WT), half of the minimum RPM (except for 0) value was added to all RPM values.

### Gene expression analysis

Total RNA was extracted from explants on day 7 of SIM (C10S7) incubation using the Monarch Total RNA Miniprep Kit (New England Biolabs, Sumida, Tokyo, Japan) following the manufacturer’s protocol. Approximately 1000 ng of total RNA was reverse transcribed using the Verso cDNA Synthesis Kit (Thermo Fisher Scientific) following the manufacturer’s protocol. The resultant cDNA was used as a template for real-time PCR with 1 or 5 times dilution. Quantitative real-time RT-PCR was performed using Luna Universal qPCR Master Mix (New England Biolabs) on a Thermal Cycler Dice Real Time System *II* (TAKARA Bio, Kusatsu, Shiga, Japan). Expression level of *PP2A* was used for the normalization of expression levels of genes of interest. The primer sets used for the analysis were as follows: 5′-TAGCACCAACACAACCGTCACA-3′ (F) and 5′-AGTTAACGTCTAAGCCCAAGGC-3′ (R) for *CUC2* (62); 5′-ACAGCTGTCATTATGCCTGAACCA-3′ (F) and 5′-GGTAGAGGAATCTAACGGTAGAGA-3′ (R) for *ESR1* (63); 5′-CTTCCAGATGGCACCACTAC-3′ (F) and 5′-GCGATGCTTATCTGGAACAT-3′ (R) for *WUS* (64); 5′-GACCAAGTGAACCAGGTTATTGG-3′ (F) and 5′-TACTCTCCAGTGCCTGTCTTCA-3′ (R) for *PP2A* (63).

### ChIP-seq

Root explants derived from WT (Col-0) and *hda19-3* seedlings were collected on day 10 of CIM (C10) and day 7 of SIM (C10S7) incubation; 0.1 g of explants was frozen in liquid nitrogen, ground into a fine powder, cross-linked, and nuclear-extracted in the nucleus isolation buffer (1% formaldehyde, 0.6% Triton X-100, and 14.4 mM 2-mercaptoethanol) with 1 mM Pefabloc SC (Sigma–Aldrich) and complete protease inhibitor cocktail (Sigma–Aldrich). The samples were sonicated using S2 or M220 focused ultrasonicators (Covaris, Woburn, MA, USA) and milliTUBE 1 ml AFA fiber (Covaris). The sonicated samples were incubated with anti-acetyl-histone H4 (Merck Millipore, Burlington, MA, USA) and antihistone H3 (ab1791; Abcam, Cambridge, UK) antibodies at 4°C overnight. Protein G Magnetic Dynabeads (Thermo Fisher Scientific) were used for immunoprecipitation. The beads were washed two times each with PBS buffer, low-salt RIPA buffer [50 mM Tris–HCl (pH 7.8), 150 mM NaCl, 1 mM EDTA, 1% Triton X-100, 0.1% SDS, 0.1% sodium deoxycholate, and 1% complete protease inhibitor (Roche Basel, Switzerland)], high-salt RIPA buffer [50 mM Tris–HCl (pH 7.8), 500 mM NaCl, 1 mM EDTA, 1% Triton X-100, 0.1% SDS, 0.1% sodium deoxycholate, and 1% complete protease inhibitor (Roche)], LNDET buffer [250 mM LiCl, 1% IGEPAL, 1% sodium deoxycholate, 1 mM EDTA, and 10 mM Tris–HCl (pH 7.8)], and with TE buffer. After adding the elution buffer [10 mM Tris–HCl (pH 7.8), 0.3 M NaCl, 5 mM EDTA, and 0.5% SDS], all beads were incubated overnight at 65°C. Lysates were treated with 200 ng mL^−1^ RNaseA at 37°C for 30 min and then treated with 800 ng mL^−1^ proteinase K and 400 ng mL^−1^ glycogen at 37°C for 2 h. After phenol–chloroform extraction and ethanol precipitation, the pellet was suspended in buffer EB (Qiagen, Venlo, Netherlands). The collected DNA was quantified using the Qubit dsDNA High Sensitivity Assay Kit (Thermo Fisher Scientific), and 1 ng DNA was used to construct a sequencing library with the KAPA Hyper Prep Kit for Illumina (Kapa Biosystems, Wilmington, USA). Dual-size selection was performed using Agencourt AMPure XP (Beckman Coulter, Brea, CA, USA) to enrich 300 to 500 bp fragments. Libraries were pooled, and 75 bp single-read sequences were obtained using the NextSeq 500 sequencer (Illumina).

Genome-wide localization patterns of the HDA19 protein were analyzed using *HDA19p::HDA19g-sGFP* transgenic plants on an *hda19-3* background and control transgenic plants expressing *35Sp:GFP* on a Col-0 background. Calli at 7 days of SIM (C10S7) incubation derived from the *HDA19p::HDA19g-sGFP* and *35Sp::GFP* plants were subjected to ChIP-seq analysis using an anti-GFP antibody (ab290; Abcam), as described above. Two independent biological replicates were analyzed for each genotype.

### ChIP-seq data analysis

Quality-filtered reads were aligned to the *Arabidopsis* reference genome TAIR10 using Bowtie (60) with -m 1 -S parameters to report only uniquely mapped reads (Data S4). The resulting SAM files were converted to sorted BAM files using SAMtools (65) and then converted to BED files using BEDTools (66). The “slop” function of BEDTools was used to extend the 5′-end of ChIP-seq reads toward the 3′-direction to fit the average insertion size (250 bp) of the sequenced libraries. Then, the “coverage” function of BEDTools was used to calculate the number of reads that overlapped with each annotation unit (Data S2). HDA19-binding sites were detected by model-based analysis of ChIP-seq data (MACS2) (67) using reads from the anti-GFP (*35Sp::GFP*) sample as controls (*q* < 0.001) (Data S3). For visualization, TDF files were created using igvtools (extension factor: 200) from BAM files and visualized using the Integrative Genome Viewer (68). The ngs.plot.r program (69) was used to determine the acetylation profiles and HDA19-binding regions of gene bodies. Scatter and NGS plots of ChIP-seq results are shown only for one of the biological replicates, because the two replicates showed very high reproducibility (Fig. S7). To calculate the acetylation level ratio (*hda19*/WT), half of the minimum RPM value was added to all RPM values.

## Supplementary Material

pgad002_Supplemental_File

## Data Availability

Data generated or analyzed during this study are included in this published article (and its supplementary data files). Data S1 contains source data corresponding to the Fig. 3C and D. Data S2 contains source data corresponding to Figs. 3A to C and 4A. Data S3 contains source data corresponding to Fig. 4C and Fig. S3. Data S4 contains the summary of RNA-seq and ChIP-seq experiments corresponding to Fig. 3. RNA-seq and ChIP-seq data that support the findings of this study have been deposited in the DNA Data Bank of Japan (Accession No. DRA13879 and DRA13880, respectively).
