# Supplementary material for: Histone deacetylation regulates de novo shoot regeneration
Source: PNAS Nexus. 2023 Jan 6;2(2):pgad002. doi: 10.1093/pnasnexus/pgad002 (PMC9944245; doi:10.1093/pnasnexus/pgad002)
Supplement: pgad002_Supplemental_File [file pgad002_supplemental_file.pdf]

PNAS nexus

Supplementary Information for

Histone deacetylation regulates *de novo* shoot regeneration

Haruka Temman, Takuya Sakamoto, Minoru Ueda, Kaoru Sugimoto, Masako Migihashi, Kazunari Yamamoto, Yayoi Inui, Hikaru Sato, Mio K. Shibuta, Norikazu Nishino, Tomoe Nakamura, Hiroaki Shimada, Yukimi Y. Taniguchi, Seiji Takeda, Mitsuhiro Aida, Takamasa Suzuki, Motoaki Seki, Sachihiro Matsunaga*

*Sachihiro Matsunaga
Email: sachi@edu.k.u-tokyo.ac.jp

This PDF file includes:

Supplementary information overview
Figures S1 to S7

Other supplementary materials for this manuscript include the following:

Data S1 to S4

Supplementary information overview

Figure S1. Shoot regeneration phenotypes in root explants of various *hdac* mutants.
Figure S2. Shoot regeneration phenotypes in root, hypocotyl, first leaf, and petal explants.
Figure S3. Expression levels of *ESR1*, *CUC2*, and *WUS* during shoot induction.
Figure S4. Dynamics of *ESR1* and *CUC2* expression during shoot induction in Ky-2 treated calli.
Figure S5. Dynamics of the expression of HDA19 target candidate genes upon shoot induction.
Figure S6. Dynamics of *WOX5* expression during shoot induction in *hda19.*
Figure S7. Reproducibility between two replicates in ChIP-seq analyses.
Data S1. Expression level of all genes and classified genes.
Data S2. Histone H4 acetylation level of all genes.
Data S3. Summary of HDA19 binding sites.
Data S4. RNA-Seq and ChIP-Seq experiment summary.

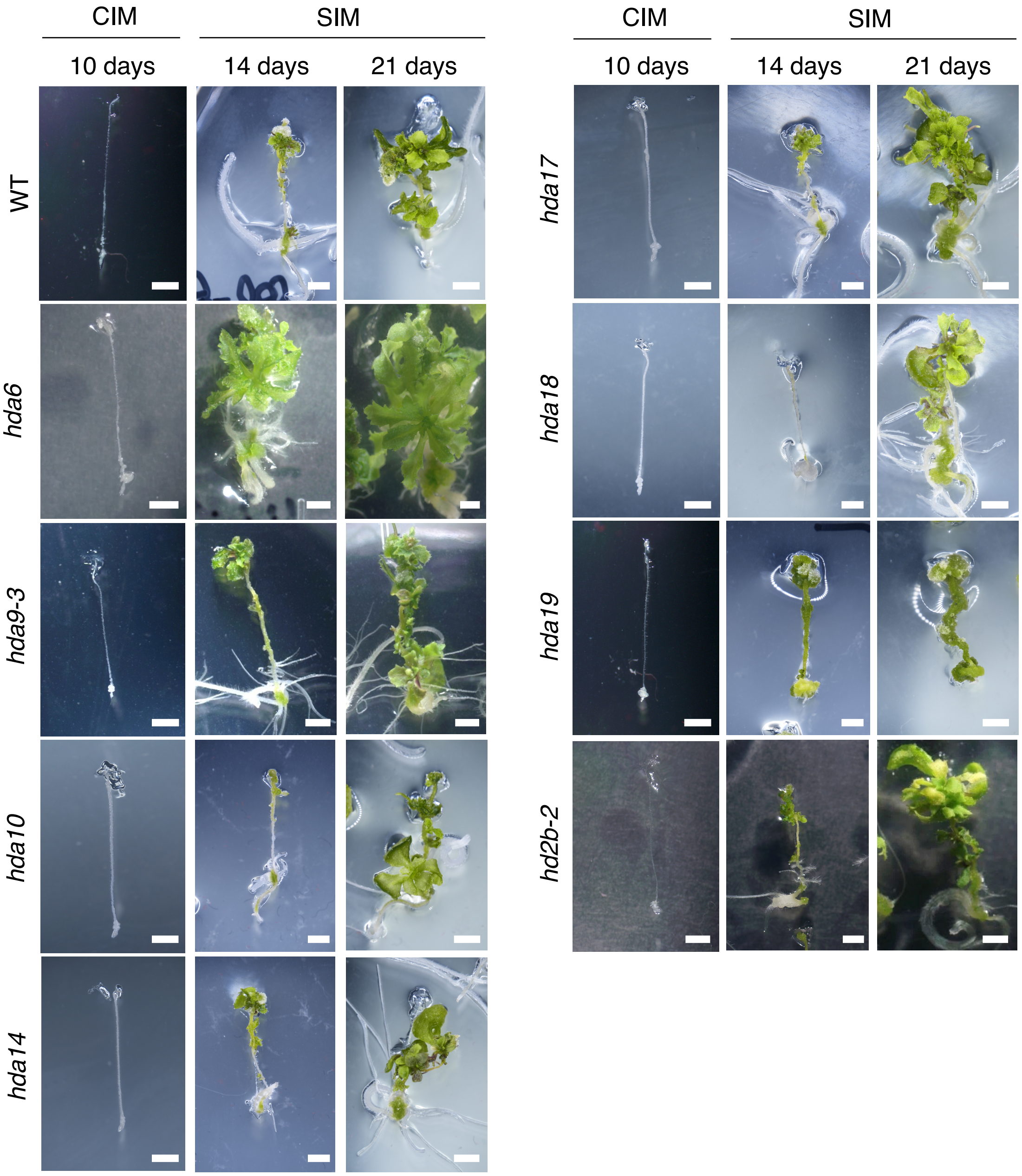

Figure S1. Shoot regeneration phenotypes in root explants of various *hdac* mutants. Phenotypes of callus formation and shoot regeneration in root explants of various *hdac* mutants at 10 days on CIM and 14 and 21 days on SIM. Scale bar = 2 mm.

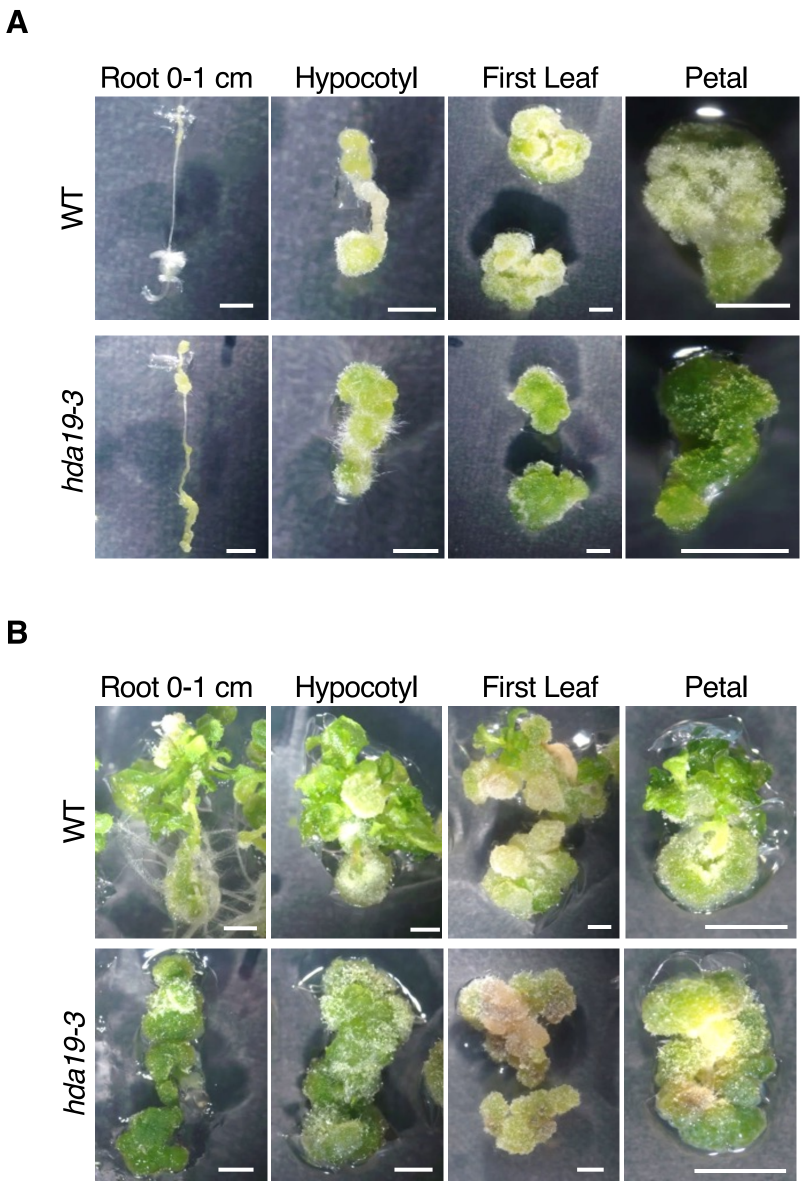

Figure S2. Shoot regeneration phenotypes in root, hypocotyl, first leaf, and petal explants. Phenotypes of callus formation and shoot regeneration in root, hypocotyl, first leaf, and petal explants of WT and *hda19* at 7 **(A)** and 21 **(B)** days on SIM. Scale bar = 2 mm.

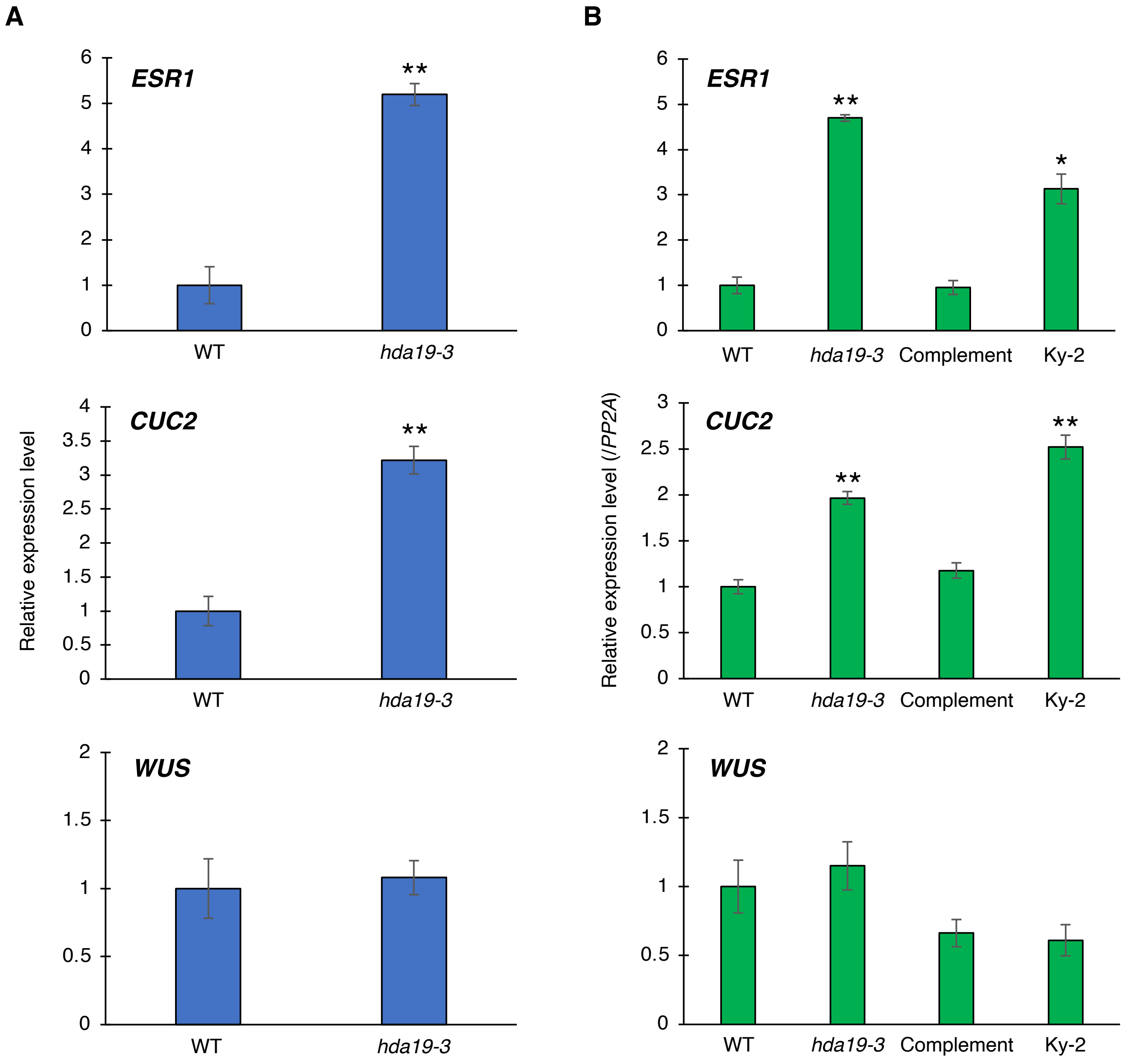

Figure S3. Expression levels of *ESR1*, *CUC2*, and *WUS* during shoot induction. **(A)** Expression level of each gene in WT and *hda19-3* explants at C10S7 determined by RNA-seq analysis was shown. **(B)** Expression level of each gene in WT, *hda19-3*, complement, and Ky-2 treated WT explants at C10S7 determined by quantitative PCR analysis was shown. Results are presented as means ± SE of three and four replicates for **(A)** and **(B)**, respectively (*$p < 0.005$, **$p < 0.001$, compared with wild type, Student's *t*-test).

A

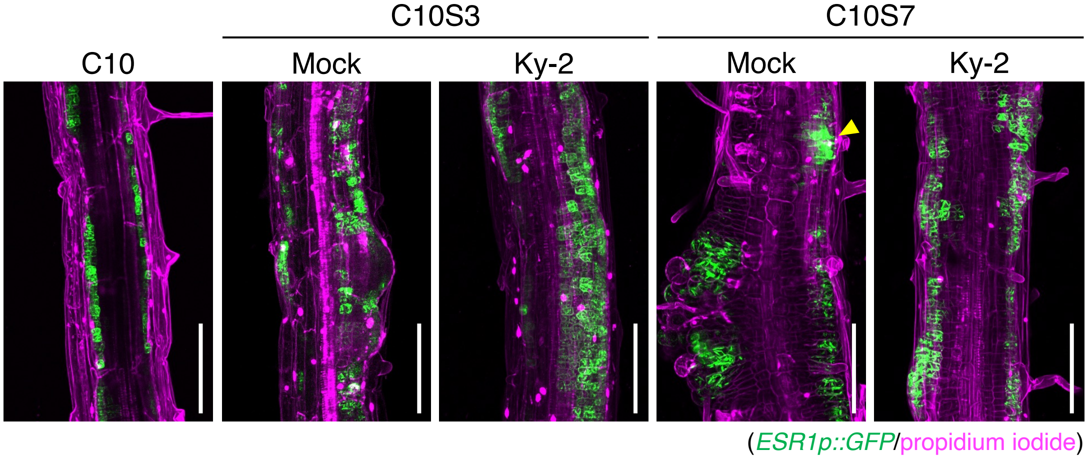

B

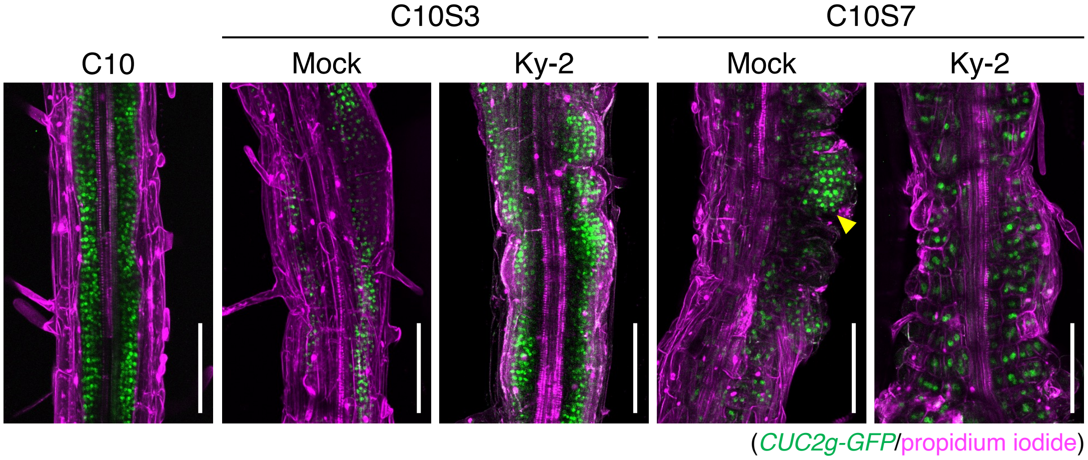

Figure S4. Dynamics of *ESR1* and *CUC2* expression during shoot induction in Ky-2 treated calli. Expression patterns of **(A)** *ESR1p::GFP* and **(B)** *CUC2g-GFP* during shoot induction in wild type treated with Ky-2. GFP fluorescence is shown in green, and the outline of PI-stained cells is shown in magenta. The images represent *z*-projections. Scale bars = 100 µm. Arrowheads indicate developing SAMs.

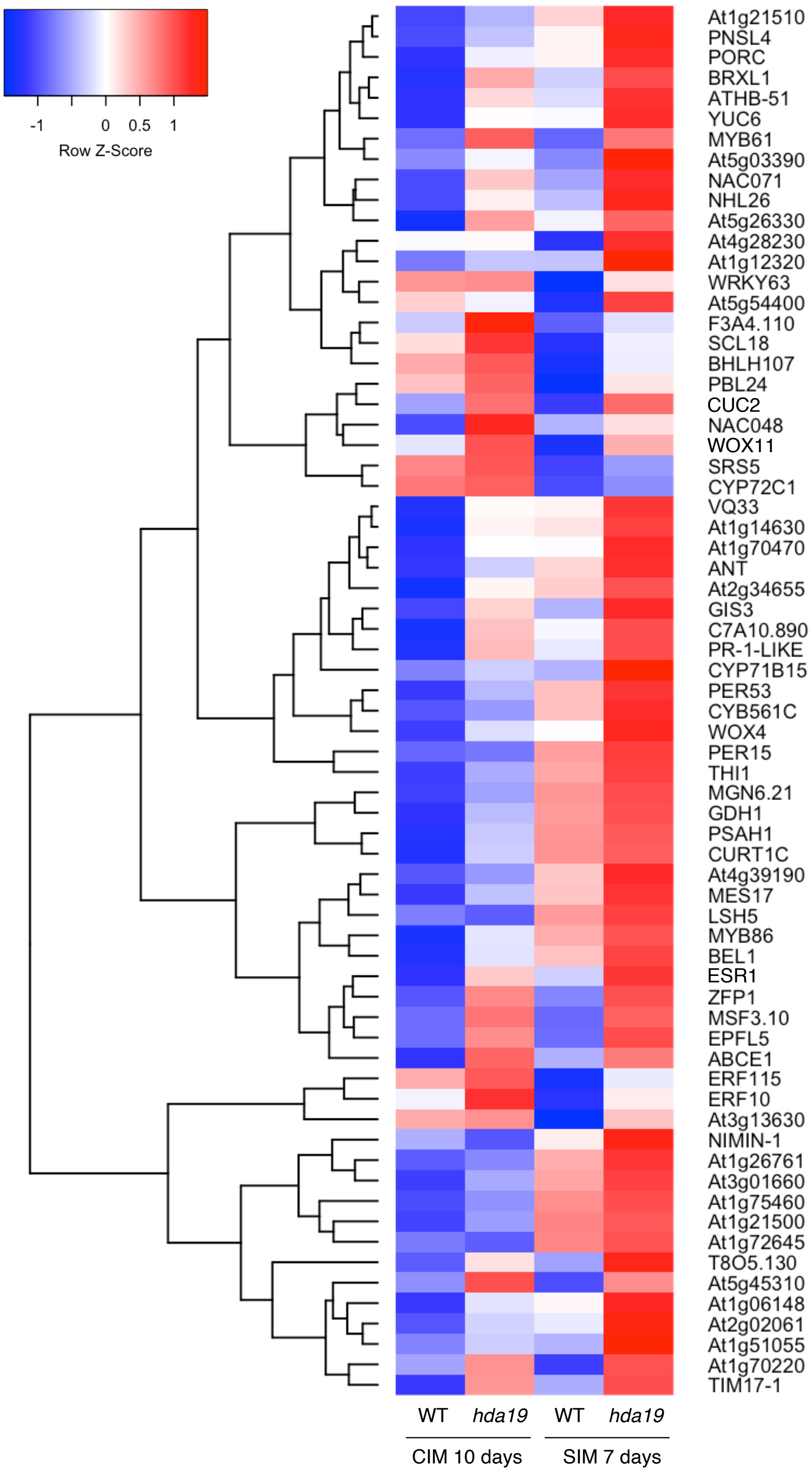

Figure S5. Dynamics of the expression of HDA19 target candidate genes upon shoot induction. Changes in the expression of HDA19 target candidate genes upon shoot induction. The heat map shows the relative expression levels of genes by the *z*-scores of read count per million mapped reads.

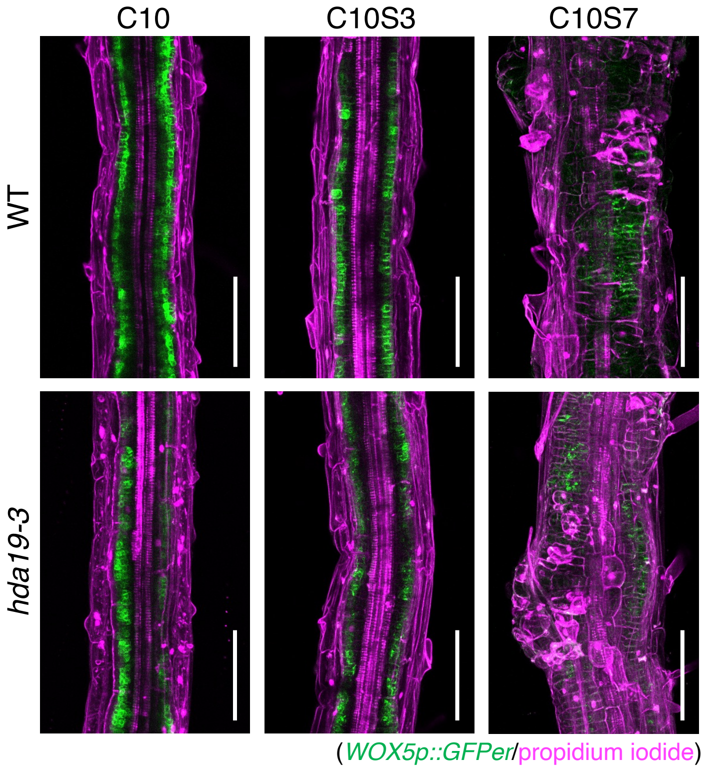

Figure S6. Dynamics of *WOX5* expression during shoot induction in *hda19*. Expression patterns of *WOX5p::GFPer* during shoot induction in *had19*. GFP fluorescence is shown in green, and the outline of PI-stained cells is shown in magenta. The images represent *z*-projections. Scale bars = 100 µm.

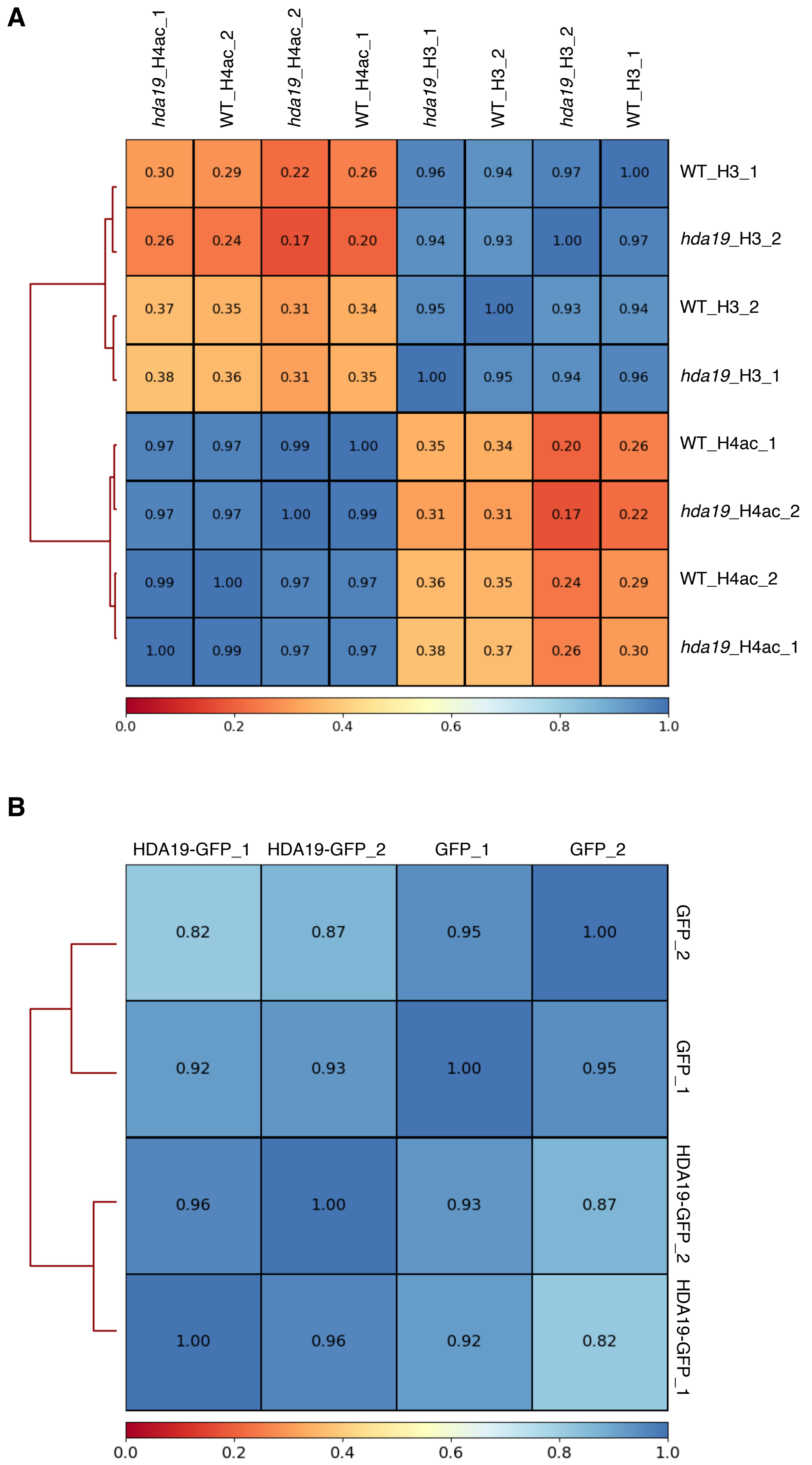

Figure S7. Reproducibility between two replicates in ChIP-seq analyses. Spearman correlation coefficients between the read counts of two samples in ChIP-seq analysis of histone modification **(A)** and HDA19 binding **(B)** were calculated.
